# 1-Methyl-1-azonia-3,5-diaza-7-phospha­tricyclo­[3.3.1.1]decane 7-oxide triiodide

**DOI:** 10.1107/S1600536808001426

**Published:** 2008-01-23

**Authors:** Alexander M. Kirillov, Piotr Smoleński, M. Fátima C. Guedes da Silva, Armando J. L. Pombeiro

**Affiliations:** a Centro de Química Estrutural, Complexo Interdisciplinar, Instituto Superior Técnico, TU Lisbon, Avenida Rovisco Pais, 1049-001 Lisbon, Portugal; b Universidade Lusófona de Humanidades e Tecnologias, ULHT Lisbon, Avenida do Campo Grande 376, 1749-024 Lisbon, Portugal

## Abstract

The title compound, C_7_H_15_N_3_OP^+^·I_3_^−^, is a derivative of the well known water-soluble amino­phosphine 1,3,5-triaza-7-phosphaadamantane (PTA). The crystal structure is composed of a cage-like 1-methyl-1-azonia-3,5-diaza-7-phospha­tricyclo­[3.3.1.1]decane 7-oxide cation and a triiodide anion. The *N*-methyl­ation of the PTA cage results in a slight elongation of the corresponding C—N bonds, while the oxidation of the P atom leads to a slight shortening of the C—P bonds in comparison with those of PTA. In general, most of the bonding parameters are comparable with those reported for related compounds bearing the PTA core. Two inter­molecular C—H⋯O hydrogen bonds between methyl­ene groups and the P=O group are responsible for the linkage of neighbouring cations into linear one-dimensional hydrogen-bonded chains.

## Related literature

For a comprehensive review of PTA chemistry, see: Phillips *et al.* (2004 ▶). For general background, see: Kirillov *et al.* (2007 ▶); Smoleński & Pombeiro (2008 ▶). For synthesis of PTA and its *N*-methyl­ated derivative, see: Daigle *et al.* (1974 ▶); Daigle (1998 ▶). For related structures, see: Forward *et al.* (1996*a* ▶,*b* ▶); Otto *et al.* (2005 ▶); Frost *et al.* (2006 ▶); Marsh *et al.* (2002 ▶).
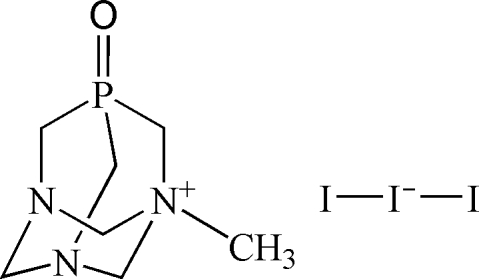


## Experimental

### 

#### Crystal data

C_7_H_15_N_3_OP^+^·I_3_^−^
*M* *_r_* = 568.89
Monoclinic, 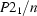

*a* = 7.1570 (8) Å
*b* = 8.2257 (8) Å
*c* = 25.903 (3) Å
β = 92.472 (7)°
*V* = 1523.5 (3) Å^3^
*Z* = 4
Mo *K*α radiation
μ = 6.24 mm^−1^
*T* = 150 (2) K
0.13 × 0.10 × 0.10 mm

#### Data collection

Bruker SMART CCD area-detector diffractometer
Absorption correction: multi-scan (*SADABS*; Sheldrick, 1996 ▶) *T*_min_ = 0.497, *T*_max_ = 0.574 (expected range = 0.464–0.536)
11526 measured reflections
2789 independent reflections
2214 reflections with *I* > 2σ(*I*)
*R*_int_ = 0.040

#### Refinement

*R*[*F* ^2^ > 2σ(*F* ^2^)] = 0.042
*wR*(*F* ^2^) = 0.102
*S* = 1.12
2789 reflections
172 parameters
H atoms treated by a mixture of independent and constrained refinement
Δρ_max_ = 2.44 e Å^−3^
Δρ_min_ = −1.03 e Å^−3^

### 

Data collection: *SMART* (Bruker, 2004 ▶); cell refinement: *SAINT* (Bruker, 2004 ▶); data reduction: *SAINT*; program(s) used to solve structure: *WinGX* (Version 1.70.01; Farrugia, 1999 ▶); program(s) used to refine structure: *SHELXL97* (Sheldrick, 2008 ▶); molecular graphics: *Mercury* (Macrae *et al.*, 2006 ▶); software used to prepare material for publication: *SHELXL97*.

## Supplementary Material

Crystal structure: contains datablocks I, global. DOI: 10.1107/S1600536808001426/kp2159sup1.cif

Structure factors: contains datablocks I. DOI: 10.1107/S1600536808001426/kp2159Isup2.hkl

Additional supplementary materials:  crystallographic information; 3D view; checkCIF report

## Figures and Tables

**Table:** 

C1—N1	1.479 (8)
C1—P1	1.821 (8)
C2—N2	1.486 (8)
C2—P1	1.799 (7)
C3—N3	1.495 (9)
C3—P1	1.825 (8)
C4—N3	1.496 (9)
C12—N1	1.462 (9)
C12—N2	1.467 (10)
C23—N2	1.440 (9)
C23—N3	1.550 (9)
C31—N1	1.441 (9)
C31—N3	1.551 (9)
O1—P1	1.483 (5)
I1—I3	2.9067 (8)
I1—I2	2.9127 (7)

**Table:** 

I3—I1—I2	172.41 (2)

**Table 2:** Hydrogen-bond geometry (Å, °)

*D*—H⋯*A*	*D*—H	H⋯*A*	*D*⋯*A*	*D*—H⋯*A*
C23—H23*A*⋯O1^i^	0.99 (10)	2.26 (11)	3.161 (9)	150 (9)
C31—H31*A*⋯O1^i^	0.97 (10)	2.23 (10)	3.160 (9)	161 (8)

Symmetry code: (i) 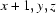.

**Fig. 1.**
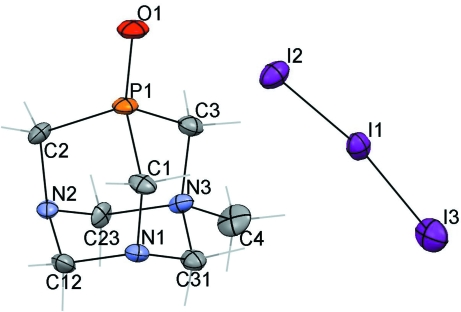
The molecular structure of the title compound with the atom labelling scheme. Displacement ellipsoids are drawn at the 50% probability level. H atoms are represented as grey sticks. C, grey; N, blue; P, orange; O, red; I, purple.

**Fig. 2.**
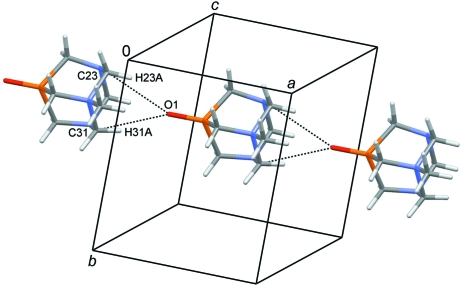
Fragment of the crystal packing diagram of (I) showing the generation of a one-dimensional linear chain from the neighbouring cations via intermolecular C—H···O hydrogen bonds (dotted lines). Tri-iodide anions are omitted for clarity. C, grey; N, blue; P, orange; O, red; H, pale grey.

## supplementary crystallographic information

### Comment

Within our ongoing research (Kirillov *et al.*, 2007; Smoleński & Pombeiro, 2008) on the synthesis of transition metal complexes with PTA or derived ligands, we have attempted the reaction of a copper^(II)^ salt with *N*-methyl-1,3,5-triaza-7-phospha-adamantane iodide, which resulted in the formation of the title compound, (I), as a by-product. Its crystal structure is reported herein.

The molecular structure of (I) (Fig. 1) bears a cage-like cation [C_7_H_15_N_3_OP]^+^ and a tri-iodide anion, with the shortest cation···anion separation of *ca* 4.0 Å. The *N*-methylation of the PTA cage results in a slight elongation of the C—N bonds around N3 atom [*avg.* 1.53 (1) Å] in comparison with the C—N bonds around N1 and N2 atoms [*avg.* 1.46 (1) Å] (Table 1). The oxidation of P1 atom also slightly affects the C—P bonds [*avg.* 1.82 (1) Å] which are somewhat shorter than those in PTA [*avg.* 1.86 (1) Å]. The tri-iodide anion with the I2—I1—I3 angle of 172.41 (2)° deviates from the linear geometry. In general, most of the bonding parameters of (I) agree within values reported for the related compound, [C_7_H_15_N_3_OP][BPh_4_] (Forward *et al.*, 1996*a*,b), possessing similar cation, as well as for other *N*-alkylated (Otto *et al.*, 2005; Forward *et al.*, 1996*a*,*b*) or *P*-oxidized (Frost *et al.*, 2006; Marsh *et al.*, 2002) PTA derivatives.

In (I), the neighbouring cationic units are combined into the linear one-dimensional H-bonded chains (Fig. 2) by means of two intermolecular C—H···O hydrogen bonds [C23—H23A···O1^i^ 1.00 (11) Å, 2.26 (11) Å, 3.161 (9) Å, 150 (9)°; C31—-H31A···O1^i^ 0.97 (10) Å, 2.23 (10) Å, 3.160 (9) Å, 161 (8)°; symmetry code: 1 + *x*, *y*, *z*], which link the methylene groups (C23, C31) with the O1 atom of the P=O moiety.

### Experimental

The aqueous solutions (5 ml each) of Cu(NO_3_)_2_.2.5 H_2_O (116 mg, 0.50 mmol) and *N*-methyl-1,3,5-triaza-7-phospha-adamantane iodide, [C_7_H_15_N_3_P]I (299 mg, 1.00 mmol) [for the synthesis of this compound, see: Daigle *et al.* (1974); Daigle (1998)], were combined and left stirring in air at ambient temperature for 1 h. The resulting white suspension containing mainly a Cu^I^ aminophosphine compound was filtered off. The colourless filtrate was left to evaporate in a beaker in air for two weeks, leading to the formation of a small crop of red X-ray quality crystals of compound (I) as a by-product (it is typically contaminated by a colourless crystalline material). FT–IR (KBr pellet), cm^-1^: 2967 w, 2939 w, 1449 m, 1384 s, 1304 m, 1279 w, 1246 w, 1195 s [ν(P=O)], 1108 w, 1091 w, 1066 w, 1019 m, 983 m, 934 m, 900 w, 876 w, 816 m, 792 w, 752 m, 544 w, 441 w, 408 w. FAB-MS^+^ (*m*-nitrobenzylicalcohol), *m*/*z*: 188 [C_7_H_15_N_3_OP]^+^.

### Refinement

All hydrogen atoms were located except from H4A, H4B and H4C which were inserted in calculated positions.

### Crystal data

**Table:**

C_7_H_15_N_3_OP^+^·I_3_^–^	*F*_000_ = 1040
*M**_r_* = 568.89	*D*_x_ = 2.480 Mg m^−^^3^
Monoclinic, *P*2_1_/*n*	Mo *K*α radiation λ = 0.71069 Å
Hall symbol: -P2yn	Cell parameters from 2835 reflections
*a* = 7.1570 (8) Å	θ = 2.6–27.9º
*b* = 8.2257 (8) Å	µ = 6.24 mm^−^^1^
*c* = 25.903 (3) Å	*T* = 150 (2) K
β = 92.472 (7)º	Plate, red
*V* = 1523.5 (3) Å^3^	0.13 × 0.10 × 0.10 mm
*Z* = 4	

### Data collection

**Table:**

Bruker SMART CCD area-detector diffractometer	2789 independent reflections
Radiation source: fine-focus sealed tube	2214 reflections with *I* > 2σ(*I*)
Monochromator: graphite	*R*_int_ = 0.040
*T* = 150(2) K	θ_max_ = 25.4º
φ and ω scans	θ_min_ = 2.9º
Absorption correction: multi-scan(SADABS; Sheldrick, 1996)	*h* = −8→8
*T*_min_ = 0.497, *T*_max_ = 0.574	*k* = −9→9
11526 measured reflections	*l* = −29→31

### Refinement

**Table:**

Refinement on *F*^2^	Secondary atom site location: difference Fourier map
Least-squares matrix: full	Hydrogen site location: inferred from neighbouring sites
*R*[*F*^2^ > 2σ(*F*^2^)] = 0.042	H atoms treated by a mixture of independent and constrained refinement
*wR*(*F*^2^) = 0.102	*w* = 1/[σ^2^(*F*_o_^2^) + (0.045*P*)^2^ + 6.2243*P*] where *P* = (*F*_o_^2^ + 2*F*_c_^2^)/3
*S* = 1.12	(Δ/σ)_max_ = 0.017
2789 reflections	Δρ_max_ = 2.44 e Å^−^^3^
172 parameters	Δρ_min_ = −1.03 e Å^−^^3^
Primary atom site location: structure-invariant direct methods	Extinction correction: none

### Special details

**Table:**

Geometry. All e.s.d.'s (except the e.s.d. in the dihedral angle between two l.s. planes) are estimated using the full covariance matrix. The cell e.s.d.'s are taken into account individually in the estimation of e.s.d.'s in distances, angles and torsion angles; correlations between e.s.d.'s in cell parameters are only used when they are defined by crystal symmetry. An approximate (isotropic) treatment of cell e.s.d.'s is used for estimating e.s.d.'s involving l.s. planes.
Refinement. Refinement of *F*^2^ against ALL reflections. The weighted *R*-factor *wR* and goodness of fit *S* are based on *F*^2^, conventional *R*-factors *R* are based on *F*, with *F* set to zero for negative *F*^2^. The threshold expression of *F*^2^ > σ(*F*^2^) is used only for calculating *R*-factors(gt) *etc*. and is not relevant to the choice of reflections for refinement. *R*-factors based on *F*^2^ are statistically about twice as large as those based on *F*, and *R*- factors based on ALL data will be even larger.

### Fractional atomic coordinates and isotropic or equivalent isotropic displacement parameters (Å^2^)

**Table:**

	*x*	*y*	*z*	*U*_iso_*/*U*_eq_	
C1	0.5522 (9)	0.4541 (10)	0.1979 (3)	0.0186 (16)	
C2	0.5510 (9)	0.1132 (8)	0.1974 (3)	0.0156 (15)	
C3	0.5535 (10)	0.2823 (10)	0.1042 (3)	0.0188 (15)	
C4	0.8586 (11)	0.2823 (11)	0.0631 (3)	0.0294 (18)	
H4A	0.9942	0.2819	0.0701	0.044*	
H4B	0.8230	0.3796	0.0432	0.044*	
H4C	0.8222	0.1851	0.0432	0.044*	
C12	0.8197 (10)	0.2815 (10)	0.2227 (3)	0.0192 (15)	
C23	0.8255 (10)	0.1301 (8)	0.1442 (3)	0.0162 (15)	
C31	0.8255 (10)	0.4347 (10)	0.1446 (3)	0.0192 (16)	
N1	0.7565 (7)	0.4292 (7)	0.1960 (2)	0.0169 (13)	
N2	0.7569 (7)	0.1334 (7)	0.1956 (2)	0.0141 (12)	
N3	0.7614 (8)	0.2828 (7)	0.1131 (2)	0.0168 (12)	
O1	0.2300 (7)	0.2825 (7)	0.1582 (2)	0.0268 (12)	
P1	0.4367 (2)	0.2827 (2)	0.16547 (7)	0.0168 (4)	
I1	0.42016 (7)	0.78153 (6)	0.088953 (18)	0.02270 (15)	
I2	0.14227 (7)	0.78170 (6)	0.16786 (2)	0.02691 (16)	
I3	0.73414 (8)	0.78543 (8)	0.02143 (2)	0.03606 (18)	
H1A	0.517 (12)	0.452 (11)	0.232 (4)	0.043*	
H1B	0.498 (12)	0.558 (11)	0.183 (3)	0.043*	
H2A	0.501 (12)	0.108 (11)	0.232 (4)	0.043*	
H2B	0.521 (12)	0.008 (12)	0.182 (3)	0.050*	
H3A	0.512 (14)	0.190 (12)	0.084 (4)	0.060*	
H3B	0.522 (14)	0.380 (13)	0.085 (4)	0.060*	
H12A	0.948 (16)	0.286 (12)	0.225 (4)	0.060*	
H12B	0.793 (14)	0.282 (11)	0.256 (4)	0.050*	
H23A	0.964 (15)	0.135 (13)	0.147 (4)	0.060*	
H23B	0.769 (13)	0.034 (13)	0.127 (4)	0.060*	
H31A	0.959 (14)	0.414 (12)	0.147 (4)	0.060*	
H31B	0.768 (13)	0.538 (13)	0.126 (4)	0.060*	

### Atomic displacement parameters (Å^2^)

**Table:**

	*U*^11^	*U*^22^	*U*^33^	*U*^12^	*U*^13^	*U*^23^
C1	0.010 (3)	0.026 (5)	0.020 (4)	0.005 (3)	0.001 (3)	−0.003 (3)
C2	0.014 (3)	0.011 (4)	0.022 (4)	−0.002 (3)	0.006 (3)	0.000 (3)
C3	0.013 (3)	0.021 (4)	0.023 (4)	0.002 (3)	−0.003 (3)	0.000 (3)
C4	0.027 (4)	0.041 (5)	0.022 (4)	−0.001 (4)	0.017 (3)	−0.007 (4)
C12	0.016 (4)	0.021 (4)	0.020 (4)	−0.001 (3)	−0.006 (3)	0.000 (3)
C23	0.018 (4)	0.006 (4)	0.026 (4)	0.004 (3)	0.005 (3)	0.002 (3)
C31	0.012 (4)	0.027 (5)	0.019 (4)	0.001 (3)	0.002 (3)	0.001 (3)
N1	0.011 (3)	0.020 (3)	0.019 (3)	−0.001 (2)	−0.002 (2)	0.000 (3)
N2	0.009 (3)	0.017 (3)	0.017 (3)	0.001 (2)	0.002 (2)	0.002 (2)
N3	0.014 (3)	0.020 (3)	0.016 (3)	−0.005 (3)	0.002 (2)	−0.004 (3)
O1	0.009 (2)	0.029 (3)	0.042 (3)	0.000 (2)	−0.002 (2)	0.002 (3)
P1	0.0075 (8)	0.0187 (9)	0.0244 (10)	0.0007 (7)	0.0003 (7)	−0.0004 (8)
I1	0.0282 (3)	0.0183 (3)	0.0215 (3)	0.0009 (2)	−0.00114 (19)	−0.0002 (2)
I2	0.0230 (3)	0.0207 (3)	0.0376 (3)	0.0000 (2)	0.0081 (2)	0.0000 (2)
I3	0.0341 (3)	0.0527 (4)	0.0218 (3)	0.0017 (3)	0.0054 (2)	−0.0023 (3)

### Geometric parameters (Å, °)

**Table:**

C1—N1	1.479 (8)	C12—N1	1.462 (9)
C1—P1	1.821 (8)	C12—N2	1.467 (10)
C1—H1A	0.94 (9)	C12—H12A	0.92 (11)
C1—H1B	1.01 (9)	C12—H12B	0.90 (11)
C2—N2	1.486 (8)	C23—N2	1.440 (9)
C2—P1	1.799 (7)	C23—N3	1.550 (9)
C2—H2A	0.98 (9)	C23—H23A	0.99 (10)
C2—H2B	0.97 (10)	C23—H23B	0.99 (10)
C3—N3	1.495 (9)	C31—N1	1.441 (9)
C3—P1	1.825 (8)	C31—N3	1.551 (9)
C3—H3A	0.96 (10)	C31—H31A	0.97 (10)
C3—H3B	0.96 (11)	C31—H31B	1.06 (10)
C4—N3	1.496 (9)	O1—P1	1.483 (5)
C4—H4A	0.9800	I1—I3	2.9067 (8)
C4—H4B	0.9800	I1—I2	2.9127 (7)
C4—H4C	0.9800		
			
N1—C1—P1	107.9 (5)	N2—C23—H23A	108 (6)
N1—C1—H1A	109 (5)	N3—C23—H23A	106 (6)
P1—C1—H1A	107 (6)	N2—C23—H23B	107 (6)
N1—C1—H1B	118 (5)	N3—C23—H23B	107 (6)
P1—C1—H1B	109 (5)	H23A—C23—H23B	117 (8)
H1A—C1—H1B	105 (7)	N1—C31—N3	110.8 (6)
N2—C2—P1	109.3 (5)	N1—C31—H31A	109 (6)
N2—C2—H2A	116 (5)	N3—C31—H31A	99 (6)
P1—C2—H2A	106 (5)	N1—C31—H31B	108 (5)
N2—C2—H2B	107 (5)	N3—C31—H31B	108 (5)
P1—C2—H2B	114 (5)	H31A—C31—H31B	122 (8)
H2A—C2—H2B	104 (7)	C31—N1—C12	110.7 (6)
N3—C3—P1	110.8 (5)	C31—N1—C1	114.0 (5)
N3—C3—H3A	112 (6)	C12—N1—C1	112.6 (6)
P1—C3—H3A	109 (6)	C23—N2—C12	110.4 (6)
N3—C3—H3B	106 (6)	C23—N2—C2	113.9 (5)
P1—C3—H3B	110 (6)	C12—N2—C2	111.2 (6)
H3A—C3—H3B	109 (8)	C4—N3—C3	111.3 (6)
N3—C4—H4A	109.5	C4—N3—C31	108.6 (5)
N3—C4—H4B	109.5	C3—N3—C31	110.7 (6)
H4A—C4—H4B	109.5	C4—N3—C23	108.0 (6)
N3—C4—H4C	109.5	C3—N3—C23	110.4 (6)
H4A—C4—H4C	109.5	C31—N3—C23	107.8 (5)
H4B—C4—H4C	109.5	O1—P1—C2	119.2 (3)
N1—C12—N2	112.4 (5)	O1—P1—C1	119.3 (3)
N1—C12—H12A	107 (6)	C2—P1—C1	101.5 (3)
N2—C12—H12A	111 (6)	O1—P1—C3	112.4 (3)
N1—C12—H12B	112 (6)	C2—P1—C3	100.5 (4)
N2—C12—H12B	113 (6)	C1—P1—C3	100.8 (4)
H12A—C12—H12B	100 (9)	I3—I1—I2	172.41 (2)
N2—C23—N3	111.1 (5)		
			
N3—C31—N1—C12	57.7 (7)	N1—C31—N3—C4	−172.0 (6)
N3—C31—N1—C1	−70.5 (8)	N1—C31—N3—C3	65.6 (7)
N2—C12—N1—C31	−59.3 (8)	N1—C31—N3—C23	−55.2 (7)
N2—C12—N1—C1	69.6 (8)	N2—C23—N3—C4	172.4 (6)
P1—C1—N1—C31	65.5 (7)	N2—C23—N3—C3	−65.7 (7)
P1—C1—N1—C12	−61.6 (7)	N2—C23—N3—C31	55.3 (7)
N3—C23—N2—C12	−57.4 (7)	N2—C2—P1—O1	174.7 (4)
N3—C23—N2—C2	68.6 (7)	N2—C2—P1—C1	−52.0 (5)
N1—C12—N2—C23	59.1 (7)	N2—C2—P1—C3	51.4 (5)
N1—C12—N2—C2	−68.4 (7)	N1—C1—P1—O1	−175.4 (4)
P1—C2—N2—C23	−64.0 (7)	N1—C1—P1—C2	51.3 (6)
P1—C2—N2—C12	61.6 (6)	N1—C1—P1—C3	−51.8 (6)
P1—C3—N3—C4	179.9 (5)	N3—C3—P1—O1	179.9 (5)
P1—C3—N3—C31	−59.2 (7)	N3—C3—P1—C2	−52.2 (6)
P1—C3—N3—C23	60.0 (7)	N3—C3—P1—C1	51.8 (6)

### Hydrogen-bond geometry (Å, °)

**Table:**

*D*—H···*A*	*D*—H	H···*A*	*D*···*A*	*D*—H···*A*
C23—H23A···O1^i^	0.99 (10)	2.26 (11)	3.161 (9)	150 (9)
C31—H31A···O1^i^	0.97 (10)	2.23 (10)	3.160 (9)	161 (8)

Symmetry codes: (i) *x*+1, *y*, *z*.
